# Report of Perineal Section

**Published:** 1892-02

**Authors:** Lucius Lamar

**Affiliations:** Reporter


### REPORT OF PERINEAL SECTION,

Performed by Dr. F. W. McRae before the Class of the Atlanta Medical College.

---

The operation for the relief of a stricture of the deep urethra "of the tortuous variety," caused by gonorrhœa. It was an old case of five years' duration, and had been treated about one year previous by gradual dilatation.

The operation was performed on October 31, 1891. The patient was a strong, healthy-looking negro named Geo. Wood, age twenty-three, occupation porter on train.

He had been taking boric acid, ten grains, three times a day for a week previously as preliminary treatment.

*Operation.*—Having anæsthetized the patient, the parts were shaven and washed with a bichloride solution, strength 1 to 2,000. He was then placed in a position for lithotomy. With an assistant to hold each knee in position, Dr. McRae, after many attempts, succeeded in passing a filiform bougie, upon which was passed a tunnelled catheter; then with a third assistant to steady the catheter, the doctor proceeded to cut down upon the end of the catheter, in the median line of the perineum, making an external opening of about an inch and a half in length, dividing carefully layer after layer, until the end of the catheter was reached, then enlarging the incision from below upward. Having his assistants to hold the wound open with retractors, with a probe-pointed tenotomy knife the doctor divided the stricture, posterior to the perineal opening, from before backward, using the filiform as a guide, until the urethra was enlarged sufficient to pass in a grooved director, which was then used to divide the stricture upon. A gush of urine followed the division of the stricture.

The division of the deep urethral stricture anterior to the primal opening was made in the same manner. That part of the stricture in the penile urethra was then divided with an Otis dilating urethrotome, first slitting the meatus with a meatotome.

The wound was then washed out with a 1 to 2,000 solution of bichloride. A rubber catheter was then introduced through perineal opening into the bladder. The wound was then dusted with iodoform, dressed with iodoform gauze, bichloride gauze and absorbent cotton, in the order mentioned.

A T bandage was then applied to hold the dressing *in situ*. A piece of rubber tubing about two and a half feet in length was then attached to the catheter and the operation was finished.

The patient rallied well from the effects of the ether. The wound was dressed again on the second day after the operation and a bougie introduced. It was also redressed on the fifth day and the catheter taken out, at which time the patient had a slight chill. No urine was passed through perineal opening after the tenth day. On the twenty-eighth day of November, just four weeks after the operation, the patient was at the college, at which time the doctor introduced a No. 33 French sound without any difficulty whatever. He then dismissed the patient with instructions to introduce a bougie occasionally.

LUCIUS LAMAR,
Reporter.

